# Updated draft genome sequence of *Streptomyces* sp. isolate H28 from the Meycauayan River, Philippines

**DOI:** 10.1128/mra.00174-25

**Published:** 2025-10-29

**Authors:** Hanna Hasmin T. Estadilla, Aroin B. Verdera, Arizaldo E. Castro, Kohsuke Honda, Marie Christine M. Obusan

**Affiliations:** 1 Institute of Biology, College of Science, University of the Philippines Diliman 172582, Quezon City, Philippines; 2 Department of Biology, College of Arts and Sciences, University of the Philippines 595041, Manila, Philippines; 3 Biological Sciences Department, College of Science, De La Salle University-Dasmariñas 93926 , Dasmariñas City, Philippines; 4 Department of Biotechnology, Graduate School of Engineering, Osaka University Suita Campus 594810, Suita, Japan; Indiana University, Bloomington, Bloomington, Indiana, USA

**Keywords:** draft genome sequence, *Streptomyces*, melanin

## Abstract

We report an updated draft genome sequence of *Streptomyces* sp. isolate H28, a melanin-producing bacterium recovered from the sediments of Meycauayan River, Philippines. The improvement in sequencing methodology is reflected in the reported statistics. The new sequence data is highly useful for future exploration given the melanogenesis of the bacterium.

## ANNOUNCEMENT

Melanin-producing isolate H28 was recovered from the sediments of the Meycauayan tributary of the Marilao-Meycauayan-Obando River System (MMORS) (14°44′14.29″N, 120°57′26.11″E) in Bulacan, Philippines—one of the world’s most polluted water systems (1, 2). Melanin production is likely the bacterium’s stress response to the environment (3–5).

For bacterial isolation, standard dilutions of 9:1 normal saline solution and one gram of collected sediments were prepared and inoculated onto growth media. Preliminary screening showed that isolate H28 can produce melanin, as evidenced by the occurrence of dark pigmentation when inoculated onto tyrosine agar media. A pure culture of isolate H28 was maintained in Trypticase Soy Agar media (30°C, 7 days incubation) (6, 7).

Genomic DNA was extracted using the Vivantis GF-1 Bacterial DNA Extraction Kit. The Sequencing library was prepared using the Illumina TruSeq Nano DNA library (350 bp insert). Sequencing was performed on a NovaSeq platform with a 100 bp paired-end setting. Initial assessment revealed a total of 15,560,742 sequence reads comprising 2,349,672,042 bases from the whole genome sequencing. Raw reads were processed using fastp (0.19.5) (8) with default quality control parameters, including adapter trimming, quality filtering, and removal of reads with low-quality bases (Q<15). After filtering, there were a total of 15,291,866 reads and 2,308,275,818 bases (97% of remaining bases are ≥Q20). Filtered reads were used for *de novo* assembly using the Unicycler pipeline (0.5.1) (9) with SPAdes as the assembly algorithm (3.15.3) (10). A k-mer size of 120 and a minimum contig length of 20,000 were used as assembly parameters. The resulting assembly was analyzed using Quast (5.3.0) (11). The whole genome assembly has a final length of 6,791,600 bp and a coverage of 340×. It has 20 contigs, with the largest being 1,237,597  bp in length. The assembly has an *N*_50_ of 517,880  bp, *L*_50_ of 4 contigs, and a GC content of 72.97%.

Average Nucleotide Identity (ANI) based on mumMER (4.0) (12) identified the bacterium to be most closely related to *Streptomyces viridosporus* NRRL 2414 (88.31% ANI) (GCA_002078235.1). BLAST+ (2.16.0) (13) revealed *Streptomyces griseomycini* JCM 4382 (GCF_014649595.1) as the closest identity (85.83% ANI).

The assembled genome was annotated using the NCBI Prokaryotic Genome Annotation Pipeline (14). A total of 6,075 genes were annotated—5,924 of which are protein-coding genes. Genes coding for putative tyrosinase and tyrosinase cofactor that are associated with melanin production were found. Melanin is formed through the action of tyrosinase, which converts tyrosine to melanin via a series of enzymatic and non-enzymatic reactions (6, 7). Melanin is one of the 18 secondary metabolite types that are encoded by the 31 biosynthetic gene clusters (BGCs) discovered in the genome (antiSMASH (7.1.0) [15]) (Table 1).

**TABLE 1:** Putative BGCs and number of regions found in the updated genome assembly of *Streptomyces* sp. isolate H28

BGCs found	No. of regions
nonribosomal peptides (NRPS)	4
furan	1
lanthipeptide class I	1
butyrolactone	1
melanin	2
type II polyketide synthase (T2PKS)	1
terpene	6
thiopeptide	1
leukocyte alkaline phosphatase (LAP)	1
NRPS-independent (NI)-siderophore	2
ribosomally synthesized and post-translationally modified peptides (RiPP)-like	3
RiPP recognition element (RRE)-containing	1
type I polyketide synthase (T1PKS)	2
indole	1
trans-acyltransferase polyketide synthases (transAT-PKS)	1
highly reducing type II PKS (HR-T2PKS)	1
triceptide	1
ectoine	1

The resequencing and reassembly were conducted to improve assembly quality reflected in the improved statistics as reported. We report higher coverage (26× to 340×), improved genome contiguity (1,181 contigs to 20 contigs), higher N50 (9,931 bp to 517,880 bp), and lower L50 (four contigs) compared to the previous draft assembly (1). Such improvement allowed a more accurate identification and annotation of BGCs, specifically those involved in the melanin metabolic pathway. The new assembly is deemed more appropriate for future use and exploration of the bacterium, given its melanogenesis and potential novelty.

## Data Availability

This whole genome sequencing and assembly project was deposited at GenBank under the accession numbers JBPVSX000000001 to JBPVSX010000020, BioSample number SAMN46394803, and Sequencing Read Archive (SRA) number SRR32141758.
